# Does decentralization of health systems translate into decentralization of authority? A decision space analysis of Ugandan healthcare facilities

**DOI:** 10.1093/heapol/czab074

**Published:** 2021-06-24

**Authors:** John Chen, Aloysius Ssennyonjo, Fred Wabwire-Mangen, June-Ho Kim, Griffith Bell, Lisa Hirschhorn

**Affiliations:** Northwestern University Feinberg School of Medicine, 420 East Superior Street, Chicago, IL 60611, USA; School of Public Health, College of Health Sciences, Makerere University, PO Box 7062, Kampala, Uganda; School of Public Health, College of Health Sciences, Makerere University, PO Box 7062, Kampala, Uganda; Makerere University, Kampala, Uganda; Ariadne Labs, 401 Park Drive, Boston, MA 02215, USA; Makerere University, Kampala, Uganda; Northwestern University Feinberg School of Medicine, 420 East Superior Street, Chicago, IL 60611, USA; Department of Medicine, Brigham and Women’s Hospital, 75 Francis Street, Boston, MA 02115, USA

**Keywords:** Decentralization, decision making, decision space, health care reform, local authority, policy research

## Abstract

Since the 1990s, following similar reforms to its general politico-administrative systems, Uganda has decentralized its public healthcare system by shifting decision-making power away from its central Ministry of Health and towards more distal administrative levels. Previous research has used decision space—the decision-making autonomy demonstrated by entities in an administrative hierarchy—to measure overall health system decentralization. This study aimed to determine how the decision-making autonomy reported by managers of Ugandan healthcare facilities (*de facto* decision space) differs from that which they are allocated by official policies (*de jure* decision space). Additionally, it sought to determine associations between decision space and indicators of managerial performance. Using quantitative primary healthcare data from Ugandan healthcare facilities, our study determined the decision space expressed by facility managers and the performance of their facilities on measures of essential drug availability, quality improvement and performance management. We found managers reported greater facility-level autonomy than expected in disciplining staff compared with recruitment and promotion, suggesting that managerial functions that require less financial or logistical investment (i.e. discipline) may be more susceptible to differences in *de jure* and *de facto* decision space than those that necessitate greater investment (i.e. recruitment and promotion). Additionally, we found larger public health facilities expressed significantly greater facility-level autonomy in drug ordering compared with smaller facilities, which indicates ongoing changes in the Ugandan medical supply chain to a hybrid ‘push-pull’ system. Finally, we found increased decision space was significantly positively associated with some managerial performance indicators, such as essential drug availability, but not others, such as our performance management and quality improvement measures. We conclude that increasing managerial autonomy alone is not sufficient for improving overall health facility performance and that many factors, specific to individual managerial functions, mediate relationships between decision space and performance.

**Key messages**

- Managerial functions that require more financial or logistical input—such as hiring and promotion of healthcare personnel—may be more susceptible to differences between *de jure* and *de facto* decision space than functions that require fewer of these inputs—such as the discipline of healthcare personnel.
- In Uganda, larger public health facilities (i.e. hospitals and level-IV health centres) report greater drug-ordering autonomy than smaller facilities (i.e. level-III and -II health centres). This indicates continuing adoption of policies that shifted Uganda’s medical supply chain from a solely ‘pull’ system to a hybrid ‘push-pull’ system.
- Although decision space for facility managers may be associated with improved essential drug availability, managerial autonomy alone is not sufficient for improving overall healthcare managerial performance.

## Introduction

### Decentralization and decision space

Starting in the latter half of the 20th century, decentralization—the transition of decision-making authority from higher to lower levels of organization control—has characterized health system reform in many low- and middle-income countries (LMICs) (Bhalla and Shotten, 2019, Marchildon and Bossert, 2018). Arguments in favour of health system decentralization highlight improvements to allocative efficiency due to decreased distance between decision-makers and their constituents as well as improved participation of community members into administrative affairs (Litvak *et al.*, 1998; Peckham *et al.*, 2015). However, public administration literature (Lægreid *et al.*, 2008; Peters, 2005) and health systems research (Barasa *et al.*, 2017; Peckham *et al.*, 2015; Jafari *et al.*, 2010) indicate ongoing tensions in decentralized systems between semi-autonomous lower-level units and the central government’s desire to maintain control. As a result, these systems are thought to be susceptible to inherent power struggles between their centre- and lower-level entities (Cammack *et al.*, 2007).

The level of decision-making authority demonstrated by entities in the administrative hierarchy—known as decision space—can be used as a proxy measure of the extent of autonomy accorded to decentralized units (Bossert, 2016; James *et al.*, 2019). Decision space has previously been a concept used to refer to the autonomy of middle- and lower-level governmental institutions with administrative oversight over healthcare facilities. In this study, we extend this concept to the individual healthcare facilities themselves.

Decision space can be allocated by the central government in legal and institutional frameworks such as government laws, policies and guidelines (*de jure* decision space). In healthcare systems, although *de jure* decision space is relatively static, susceptible only to changes in policies and legal instruments, *de facto* decision space—or the actual decision space reported by decentralized decision-makers—is more dynamic and multifaceted. Contextual factors such as the competence of facility managers, their knowledge of their own *de jure* decision space, the willingness of these managers to exercise discretion to push the boundaries of the *de jure* decision space they are given and managerial corruption may influence *de facto* decision space and result in differences between the actual and intended autonomy of decentralized decision-makers (Bossert, 1998; 2016; Barasa *et al.*, 2017).

Therefore, accurate measurement of *de facto* decision space is an essential first step in assessing how successfully a country’s policies of decentralization have been adopted (Cahyaningsih and Fitrady, 2019; Park *et al.*, 2013). In addition, such an assessment forms a basis to leverage decentralization to improve health system performance. As managerial autonomy increases, managers are thought to be emboldened to take more proactive steps in capacity building, operations management and other activities that improve performance (Miah and Mia, 1996). For example, Bossert and Mitchell (2011) found ‘spillover’ effects of decision space on institutional capacity, noting that increased decision space in one managerial domain tended to be associated with increased capacity in others. However, it is also possible that, without existing mechanisms to ensure accountability, increasing managerial autonomy may actually result in poorer health system performance due to less oversight over unskilled or corrupt managers (Yilmaz *et al.*, 2008; Roman *et al.*, 2017; Bossert, 2015). Therefore, further research is needed to better understand the association between managerial autonomy and health system performance.

### Decentralization in Uganda’s health system

Generally, Uganda is characterized by a decentralized politico-administrative system comprising semi-autonomous districts and lower-level councils such as municipalities, town councils and village local councils (Green, 2008; Hizaamu, 2014). Uganda’s health systems have undergone an extensive period of similar decentralization reforms. While its national Ministry of Health (MOH) still retains responsibility for national policy formulation and planning, it devolved many functions to district-level administrative units, such as operation of health centres and village health teams (Tashobya *et al.*, 2018). With respect to governance and management, facility-level entities, such as health unit management committees and hospital boards, were assigned the task of overseeing the general administration of their respective health centres or hospitals. District-level entities, such as district health management teams (DHMTs), were given executive control over service delivery and health facility performance (Tashobya *et al.*, 2018). District service commissions are tasked with human resources planning and performance management (PM) responsibilities, such as dismissal, recruitment and promotion of health workers (Public Service Commission Act, 2008). However, the actual, *de facto* decision space of these governance bodies reportedly differs from their supposed, *de jure* decision space dictated by MOH policies (Alonso-Garbayo *et al.*, 2017; Henriksson *et al.*, 2017; Tashobya *et al.*, 2018).

Additionally, facility autonomy in Uganda’s healthcare system varies by facility type and level. In Uganda, the lowest healthcare level is occupied by village health teams, followed by health centres (II, III and IV), district hospitals, regional referral hospitals and national referral hospitals. As one moves up this healthcare facility hierarchy, the complexity of care and institutional capacity of each facility type increase accordingly, as does the level of autonomy each is afforded (Mukonzo *et al.*, 2013).

Uganda also has an extensive network of private healthcare facilities, made up of for-profit healthcare providers and faith-based, not-for-profit health facility networks. The private health providers have more decision-making discretion and function generally with limited control from the public sector except for regulatory obligations (Ministry of Health, 2010). The private not-for-profits are largely autonomous—receiving most of their revenue from out-of-pocket expenditures or development partners—but still receive some financial and in-kind resource contributions from the government in exchange for meeting specified health objectives (Ssennyonjo *et al.*, 2018).

To date, research has focused on the measurement of decision space in high- to middle-level administrative units (i.e. on the level of central government planners to district administrators). Measurement of decision space for managers of individual health facilities within a health system is important for understanding the success of decentralization in levels of the healthcare administrative hierarchy more proximal to healthcare delivery. Therefore, we used data from the Performance Monitoring and Accountability (PMA) 2020 (now Performance Monitoring for Action) survey, which included an assessment of the management of primary healthcare (PHC) facilities in Uganda to answer two questions: (1) the degree to which *de facto* decision space for health facility managers differs from their intended *de jure* decision space and (2) whether an association between *de facto* decision space and managerial performance outcomes is observed at the facility level.

## Methods

### Survey platform

Data were collected through the PMA 2020 survey platform, which has gathered family planning information from households and health facilities in 11 LMICs (Pmadata.org, 2020). In Ghana and Uganda, additional sections were added to assess PHC capacity and delivery and facility management practices. For the purposes of this study, only health facility data from Uganda were used.

### Management questions

Questions on management practices were derived from the World Management Survey (WMS), a validated framework for assessing managerial performance across sectors (Bloom *et al.*, 2017a; 2020). Since the WMS was originally an intensive, open-ended, qualitative survey that was challenging to implement at scale in LMIC PHC facilities, a close-ended, quantitative version of the survey was previously developed and validated in Ghana (Macarayan *et al.*, 2019; Uy *et al.*, 2019).

### Variables

Eight multiple-choice questions—referred to as ‘authority level’ questions—were selected from the PMA survey to measure two aspects of facility autonomy: (1) decision-makers responsible for specific facility decisions and (2) the overall *de facto* decision space of facility managers.

**Figure 1.**
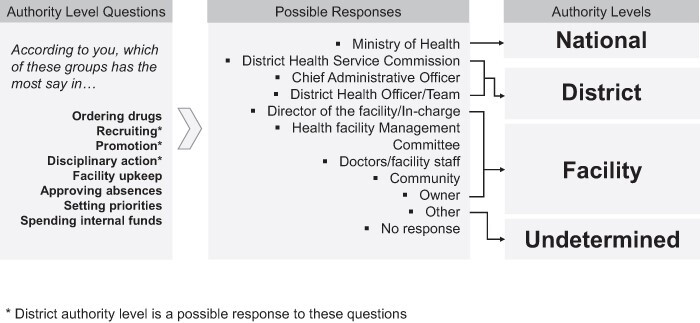
Methodology for Grouping Questionnaire Responses into Authority Levels.

These questions started by posing different situations that could arise in everyday health facility administration (hiring personnel, disciplinary action, promotion, drug ordering, facility upkeep, approving absences, setting priorities and spending funds). Respondents were then provided with a list of decision-makers and were asked to indicate which one had the most influence in responding to each respective situation. After data were gathered, responses were then grouped into four discrete authority levels (Figure 1): (i) national-level authorities (MOH), (2) district-level authorities (e.g. District Health Service Commission), (3) facility-level authorities (e.g. doctors/facility staff) and (4) undetermined authorities (‘no response’). Answers of ‘other’ were grouped separately from these authority levels. During the development of the survey in Uganda, district-level authority responses were included only for authority-level questions pertaining to human resources management (hiring, disciplinary action and promotion), as the administrative purview of Ugandan district service commissions is restricted to human resources management (Public Service Commission Act, 2008). Two variables were derived from these authority-level questions:

1. **Facility authority for specific decisions**: This was a categorical variable that, for each authority-level question, indicated whether the specific managerial decision was carried out by facility-level authorities or non-facility-level authorities (e.g. national, district, undetermined and other authorities).
2. **Overall facility authority score**: This was an ordinal variable used as a measure of overall self-perceived *de facto* decision space for health facility administrators, with a minimum possible score of 0 and a maximum possible score of 8. This was the total number of times a respondent attributed managerial decisions posed by authority-level questions to facility-level authorities.

Associations of overall facility authority scores with three indicators of managerial performance were explored using data available in the PMA PHC facility questionnaire (**S1**). The domains of managerial performance included:

- **Essential Drug Index**: It is a proportion of availability of up to 20 drugs deemed essential by the Ugandan MOH, which was adapted from the Service Delivery Indicators’ list of essential drugs and a similar index used in Ghana (Macarayan *et al.*, 2019). For the Ugandan version of the index, Ugandan MOH essential drug guidelines were consulted to determine what drugs were considered essential (Ministry of Health, 2016).
- **Quality Improvement (QI) Index**: It comprises 13 multiple-choice, Likert-scale and yes–no items assessing adherence to QI guidelines set out by the Uganda MOH (Ministry of Health, 2015a; 2019). Answers to these questions were summed and then divided by the maximum observed score (10.75) to create a ratio score from 0 to 1.
- **PM** **Index**: It comprises six multiple-choice and yes–no items assessing adherence to PM activities deemed as best practice by guidelines set out by the MOH (Ministry of Health, 2015b). Answers to these questions were summed and then divided by the maximum observed score (6) to create a ratio score from 0 to 1.

### Sampling and data collection

A multi-stage cluster sample design was used to probabilistically select enumeration areas (EAs) stratified by urban and rural areas, each of which contained approximately 200 households. Public facilities with catchment areas that overlapped with the boundaries of an EA and up to three randomly chosen private facilities within an EA were eligible to be sampled. In Uganda’s formal healthcare system, patients are referred to facilities of ascending complexity and capacity, depending on the severity of their conditions. Level-II health centres are the smallest facilities, providing basic preventative outpatient care, while national referral hospitals are the largest, offering a full range of inpatient and outpatient services (Institute for Health Metrics and Evaluation, 2014). Only public and private facilities that feed into this formal referral system were included in this study (e.g. hospitals and health centres). Therefore, chemists, pharmacies and private health clinics (which do not participate in this referral system and are distinct from private health centres) were excluded (Uganda Legal Information Institute, 2016). Decision space and managerial performance data were collected using a questionnaire, with a trained enumerator eliciting responses from a primary respondent for each health facility. Eligible respondents included the medical director, medical superintendent, or director of nursing of a public hospital or nurse-, midwife- or physician-in charge of a health centre, and the owner, managing partner, administrator or the highest-ranking doctor of a private facility. Facilities without an eligible respondent present at the time of questionnaire administration were excluded. Overall, 250 of 398 sampled facilities were included in our analysis (Table 1, Table S2).

**Table 1.** Descriptive statistics of health facilities in sample

Characteristics		Facilities (*N* = 250)	
Region		*N*	%
Central		55	22.0
Eastern		75	30
Northern		61	24.4
Western		59	23.6
Urban/Rural	Facility type	*N*	%
Urban	Hospital	9	3.6
	Health centre		
	Level IV	3	1.2
	Level III	12	4.8
	Level III	8	3.2
Rural	Hospital	38	15.2
	Health centre		
	Level IV	56	22.4
	Level III	69	27.6
	Level III	55	22.0
Ownership		*N*	%
Public		224	89.6
Private		26	10.4
Facility size		Mean	Range
Hospital	Health centre	159	0–400
	Level IV	38	0–120
	Level III	0	
	Level II	0	

### Analysis

#### Descriptive statistics

Cronbach’s alpha—a measure of scale quality that assesses internal consistency of questionnaire items—was calculated for the eight authority-level questions and managerial indicators. Overall facility authority scores and answers to individual authority-level questions were stratified by facility type and authority level, respectively, and graphically depicted. Descriptive statistics for overall facility authority scores and answers to authority-level questions were also assessed using counts and percentages. Chi-square tests were used to determine between-group differences on responses to specific authority-level questions.

#### Associations between managerial performance indicators and overall facility authority scores

Ordinary least squares (OLS) regression was used to predict the effects of our exposure of interest on our three outcome measures based on changes in overall facility authority scores. Based on previous studies using the PMA PHC facility data, the variables of public/private ownership, region, urban/rural distribution and facility type were included as covariates in our models (Macarayan *et al.*, 2019; Uy *et al.*, 2019). An interaction term between urban/rural and facility type was included as a covariate as well due to documented disparities in institutional capabilities of Ugandan hospitals, which have been attributed to whether these hospitals were located in urban or rural locations (Bossert and Beauvais, 2002). Bonferroni’s correction was used for each model to account for multiple comparisons. As each model tested 12 hypotheses, Bonferroni’s correction gave us a new significance cutoff of α = 0.004. Our outcome variables measuring QI and PM were reflected and log-transformed in order to account for their negative skewness and non-normal distributions. Predicted values for outcome measures based on our OLS models were then obtained at 90% and 10% percentiles of overall facility authority scores to determine differences in managerial performance associated with higher and lower levels of local autonomy within our sample.

## Results

### Decision space of local facility managers

Out of the 250 facilities included in our analyses, 47 (18.8%) were national referral, regional referral or general hospitals; 59 (23.6%) were level-IV health centres; 81 (32.4%) were level-III health centres and 63 (25.2%) were level-II health centres. Most (224, 89.6%) facilities were publicly owned while some (26, 10.4%) were privately owned (Table 1).

Regarding personnel management, public facility managers reported that decisions of recruitment and promotion were largely made at the district level (recruitment = 81.7%; promotion = 85.3%). However, a smaller proportion of public facility managers reported that decisions of discipline were made at the district level (58.9%), with almost a third reporting instead that facility-level authorities were responsible for these decisions (37.1%). No between-group difference for disciplinary autonomy was observed among different types of public facilities (χ^2^ = 3.53, *P* = 0.316). However, significant between-group differences were observed between public and private Ugandan healthcare facilities on autonomy in discipline (χ^2^ = 29.07, *P* < 0.001), recruitment (χ^2^ = 127.00, *P* < 0.001) and promotion (χ^2^ = 85.37, *P* < 0.001). Private facility managers largely reported greater human resources autonomy compared with their public counterparts, indicating that decision-making authority for discipline (92.3%), promotion (65.4%) and recruitment (73.1%) rested at the local level.

**Figure 2.**
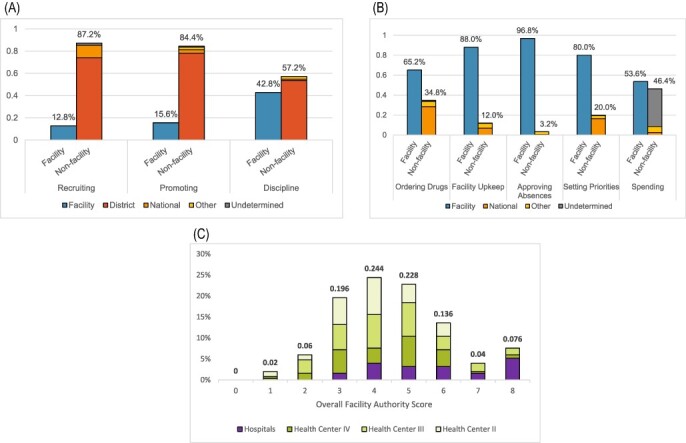
(A) Distributions for authority-level questions assessing personnel management. (B) (middle) Distributions for non-personnel management authority level questions. (C) Distributions of overall facility authority scores.

For most other decisions assessed by the authority-level questions (facility upkeep, setting priorities, approving absences, ordering drugs and spending funds), a majority of both public and private facility managers reported that these were made by facility-level authorities (facility upkeep = 88%, setting priorities = 80%, approving absences = 96.8%, ordering drugs = 65.2% and spending funds = 53.6%; Figure 2A and B).

Although the majority of facilities (65.2%) indicated autonomy over drug ordering, this was affected by the variables of facility type and public/private ownership. Significant between-group differences were seen in drug-ordering autonomy based on facility type (χ^2^ = 33.94, *P* < 0.001), with 89.4% of hospitals indicating facility-level authority over drug ordering, 76.3% of level-IV health centres, 54.32% of level-III health centres and 50.1% of level-II health centres. This between-group significance persisted when comparing the drug-ordering autonomy of hospitals and level-IV health centres vs level-III and -II health centres, indicating that the former had significantly higher drug-ordering autonomy (χ^2^ = 25.11, *P* < 0.001). No significant between-group differences in drug-ordering autonomy were seen when comparing hospitals with level-IV health centres (χ^2^ = 3.34, *P* = 0.19) and level-III with level-II health centres (χ^2^ = 3.96, *P* = 0.265). Additionally, significant between-group differences in drug-ordering autonomy were observed between public and private facilities (χ^2^ = 9.88, *P* = 0.02), with almost all private facilities (92.3%) indicating autonomy over drug ordering compared with 62.1% of public facilities.

Finally, a majority (53.6%) of facility managers reported that facility-level authorities spent internally generated funds. Private facilities were much more likely to indicate spending autonomy (96.2%) than public facilities (48.7%). Of these public facilities, hospitals were significantly more likely to report autonomy over spending (72.7%) than level-IV, -III and -II health centres (43.1%, 47.9% and 41.7%, respectively) (χ^2^ = 18.08, *P* = 0.034).

The maximum overall facility authority score reported was 8, while the minimum overall facility authority score reported was 1 (minimum possible = 0). Overall, the overall facility authority score distribution was bimodally distributed [median = 4, interquartile range (IQR) = 3–6; Figure 2C]. This bimodality was mostly due to private hospitals, which tended to report higher overall facility authority scores (median = 8, mean = 7.5) than the public hospitals or health centres. Conversely, overall facility authority scores for all health centres and public hospitals were more normally distributed (median = 4, mean = 4.25). The eight authority-level questions demonstrated acceptable internal consistency (α = 0.6021).

### Associations of local autonomy with managerial performance measures

Median Essential Drug Availability (ED), PM, and QI Index scores were 0.69, 0.87 and 0.78 (out of 1) respectively. Total score distributions for our indicators of managerial performance tended to be negatively skewed (median_ED_= 0.688, IQR_ED_ = 0.375–0.85; median_PM_ = 0.87, IQR_PM_ = 0.67–0.98; median_QI_ = 0.78, IQR_QI_ = 0.53–0.86). Cronbach’s alpha for our ED, PM and QI Indices were 0.89, 0.69 and 0.85, respectively, indicating average to good internal consistency.

After Bonferroni’s correction (α = 0.004) and controlling for the covariates of public/private ownership, region, urban/rural distribution, facility type and the interaction between urban/rural and facility type, higher overall facility authority scores were associated with higher ED (*P* = 0.002) (**S3**). Our model predicted that facilities with overall facility authority scores in the 90th percentile would have 21.3% more essential drugs available at a given time than those with facility-level autonomy scores in the 10th percentile (Table 2). Furthermore, for our authority-level question assessing specifically drug-ordering autonomy, two-sample independent *t*-tests revealed that facilities which attributed drug-ordering autonomy to facility-level authorities had significantly higher ED (mean = 0.656, SD = 0.288) compared with facilities that did not [mean = 0.509, SD = 0.290; *t*(248) = 3.83, *P* < 0.001]. We also found that hospitals and level-IV health centres, which are able to individually requisition drugs from the National Medical Stores (NMS), had higher ED (mean = 0.775, SD = 0.233) than level-III and -II health centres (mean = 0.480, SD = 0.276), which receive predetermined essential drug kits from the NMS [*t*(248) = 8.92, *P* < 0.001].

**Table 2.** Predicted scores for outcome measures at 90th and 10th percentiles of authority level scores indicating facility autonomy based on OLS regression models

	Level of facility autonomy				
	90th Percentile		10th Percentile		Relative (%) change in score
	Predicted	SE	Predicted	SEM	
Essential drug availability	0.678	0.026	0.559	0.019	21.3%^a^
Quality improvement	10.5	0.064	10.4	0.046	1.4%
Performance management	6.29	0.059	6.38	0.050	−1.4%

Level of facility autonomy measured via overall facility authority scores.

a Association is significant at α = 0.004 (after accounting for multiple comparisons).

After controlling for multiple comparisons, associations between overall facility authority scores and our indicators measuring QI (*P* = 0.042) and PM (*P* = 0.375) did not reach significance. Our models also found that the covariate of facility type was significantly associated with outcome measures. Specifically, level-II health centres were associated with lower ED compared with hospitals (*P* < 0.001). Public vs private ownership was not found to be significantly associated with managerial indicators.

## Discussion

This study, through a decision space analysis of individual Ugandan healthcare facilities, has illustrated the current status of healthcare devolution in the country and has shed light upon the broader relationship between managerial autonomy and health facility performance.

Current *de jure* policies in the Ugandan healthcare system indicate that general administrative tasks and healthcare delivery should be carried out by facility-level entities, with executive control and oversight from district-level entities (i.e. DHMTs) (Tashobya *et al.*, 2018). District-level entities are also meant to be responsible for human resources planning and PM responsibilities, such as recruiting, promoting and disciplining staff (Public Service Commission Act, 2008). Our study mostly found concordance between these *de jure* policies and the *de facto* autonomy reported by health facility managers, which is notable when considering that successful devolution of authority from central to peripheral decision-makers has not always been observed in decision space research, even in countries with ongoing decentralization efforts (Kigume *et al.*, 2018; Mohammed *et al.*, 2016). However, several exceptions did exist. For example, although public facilities indicated district-level entities had authority over recruitment and promotion, some of these facilities had taken on *de facto* control of disciplinary decisions. This could be because recruitment and promotion of health workers are more subject to financial constraints compared with discipline. Alonso-Garbayo *et al*. (2017) found that Ugandan DHMTs felt similarly constrained in making decisions of recruitment and promotion as, although they had a high level of autonomy in forecasting health sector staffing needs, they had more difficulty in receiving authorization from district governments to act upon their predictions due to budgetary restrictions. However, discipline—or firing of health workers—would result in a decrease in a health system’s financial burden, as opposed to recruitment and promotion, which would require input of financial or logistical resources. As a result, district authorities may be more likely to approve facility requests for discipline (compared with recruitment and promotion). This may lead to facility managers having higher perceived *de facto* disciplinary autonomy than expected. In Uganda, the inability of facility managers to hire and fire staff has been recognized as a constraining factor for health facility performance. Recent health system innovations characterized by performance-based financing have been thereby implemented in both private and public facilities. These measures aim to financially empower facilities to recruit personnel while bypassing public service ceilings and regulations instituted by the MOH (Ssengooba *et al.*, 2015; Renmans *et al.*, 2017).

Our study found that hospitals and level-IV health centres expressed higher facility-level drug-ordering autonomy compared with the smaller level-III and -II health centres. These findings are in line with *de jure* policies that govern the Ugandan medical supply chain. In addition to greater autonomy, the literature also reports that managers of larger public facilities are likely to have greater resources and therefore greater capacity to make use of this autonomy compared with managers of smaller facilities (Bossert and Mitchell, 2011; Marcheldon and Bossert, 2018). Public hospitals and level-IV health centres can independently requisition drugs from the NMS—the Ugandan government’s central medical supplies distributor. This is because they are assumed to be large enough to have the human resources and technical capacity to manage their own supply chains. The smaller and usually more rural level-III and -II health centres, conversely, are given predetermined packages of essential drugs by the NMS based on their projected demand (Bukuluki *et al.*, 2013; Ministry of Health, 2011). These policies were originally implemented to shift Uganda’s medical supply chain from solely a ‘push’ system, where all facilities were responsible for quantifying their own drug needs, to a hybrid ‘push-pull’ system, where facilities either manage their own supply chains or delegate this responsibility upwards to more central administrative levels. Studies have shown that while some level-III and -II health centre personnel believe these changes have improved efficiency of drug delivery, others claim that the unique consumption needs of their specific areas are not taken into account enough by central planners, resulting in stock-outs of highly essential drugs (i.e. antibiotics and anti-malarials) and excess stock of less essential drugs (i.e. anti-diarrhoea drugs) (Bukuluki *et al.*, 2013). These concerns are corroborated by our own results, as our models found that level-II health centres were associated with lower ED compared with hospitals. Therefore, while our data indicate that Uganda’s shift towards a hybrid ‘push-pull’ medical supply-chain model is undergoing adoption by individual healthcare facilities, it also suggests that smaller facilities may be more susceptible to essential drug stock-outs due to decreased supply-chain responsiveness to changes in local demand.

Our study found that, among public facilities, hospitals were more likely to indicate facility-level autonomy over the spending of internally generated funds than health centres. Since the abolition of user fees in Uganda in 2001, most of the revenue generated by public health facilities should come from external sources, such as the MOH or development partners (Ministry of Health, 2017). An exception can be seen in public hospitals, however, as after the abolition of user fees, many public hospitals established private wings as a way to bolster their revenues outside of grants from the MOH or non-governmental organizations (Ministry of Health, 2010). These private wings operate outside of the Ugandan public healthcare system and provide faster care and greater privileges for patients able to afford these services. Our finding that 72.7% of public hospital managers reported facility-level autonomy over internally generated funds could thereby be contextualized as the proportion of public hospitals that utilize this additional revenue stream. However, our study also found that nearly half (44.5%) of public health centres attributed spending of internally generated funds to facility-level authorities, despite the lack of private wings in these facilities—meaning they should not generating internal funds in the first place (Ministry of Health, 2017). This indicates potential confusion from some respondents over the distinction between internally generated funds (earned on the level of the facility) and total revenue, which can be composed of both internally generated funds and funding from district governments and development partners.

Our study was also able to further characterize the relationship between managerial autonomy and health system performance. Controlling for facility characteristics, overall facility authority scores—our measure of *de facto* decision space for individual facilities—were positively associated with ED. Furthermore, facilities that indicated greater autonomy over drug ordering had significantly higher ED than those that did not. These findings elaborate upon previous research establishing synergistic relationships between *de facto* decision space for district-level administrators and institutional capacity in Pakistan, demonstrating a similar relationship in Uganda between *de facto* decision space for lower-level, facility managers and ED, which can be seen as a possible outcome of improved capacity (Bossert and Mitchell, 2011). However, no significant associations were found between *de facto* decision space and our measures of PM and QI. This suggests that the relationship between managerial autonomy and health system performance is multifaceted and complex. Although increasing decision space may be associated with improvements in one managerial function, these improvements may not carry over to other functions. This difference is consistent with conclusions from previous decision space literature. Bossert *et al*. (2007) found that in drug-ordering supply chains in Ghana and Guatemala, increased decision space was associated with poorer performance in some functions such as inventory control, while it was associated with improved performance in other functions such as planning and budgeting. The authors hypothesized that this difference may be explained by other factors that mediate the relationship between decision space and performance, such as managerial competence and responsiveness. Liwanag and Wyss (2018) found that in the Philippines, which has been undergoing devolution for the past 25 years, most local-level public health decisions are made by elected local officials—politicians who may not have expertise in health system administration, instead of local health officers—physicians who are the *de jure* authorities on the health sector. This was reported by study participants as a hindering factor in healthcare delivery. Similarly, in Uganda, our study found greater ED in larger hospitals and health centres, whose administrators can independently requisition their own drugs in accordance with facility need, compared with smaller health centres, which receive predetermined essential drug kits from the NMS. Therefore, our findings may reinforce the importance of factors specific to managerial functions, such as ability to respond to changes in essential drug need, in mediating the association between decision space and performance of managerial functions.

There were a number of limitations to our study. First, since this study exclusively used cross-sectional data, outcomes that were more susceptible to daily fluctuations in supply, such as ED, may be more prone to error. Additionally, this meant that only inter-facility comparisons of *de facto* decision space could be made, restricting our analysis from assessing the possible effects of changes in intra-facility *de facto* decision space on managerial performance. Second, due to the design of the PMA PHC questionnaire, participants were not able to clarify responses of ‘other’ to authority-level questions. Therefore, the particular authority level these responses specify remains unclear. However, the proportion of respondents that answered ‘other’ for authority-level questions was relatively marginal (0% to 6%; Figure 2A and B). Therefore, we predict that this ambiguity did not significantly impact authority-level distributions for individual questions or overall facility authority scores. Third, this study only assessed primary decision-makers responsible for facility actions. As a result, action necessitating equal input from multiple authority levels have been simplified in our analysis. Additionally, it is possible that our questionnaire is not sensitive to discrepancies in *de facto* and *de jure* decision space due to inadequate respondent knowledge (i.e. a respondent at a public health centre believes recruitment decisions are made on a facility level when in fact they are made at a district level). A follow-up survey asking the districts themselves whether they have *de facto* control over the functions attributed to them by facility managers in this study is needed. Finally, although items from the survey used in this study have been previously validated for use in Ghana, they have not yet been subsequently validated in Uganda. Additionally, the items used for our authority-level questions have not yet been formally validated in LMICs; however, our preliminary validation of these items showed that they demonstrated acceptable internal consistency. It should also be noted that this study’s outcome measures are meant to capture quality of healthcare management, rather than quality of healthcare delivery. Previous research has shown that managerial indicators, while useful, are sometimes incongruous with the actual quality of care delivered at a health facility (Komakech, 2005).

## Conclusion

Our study has shown that decision space analysis, traditionally used to determine the autonomy of middle- and lower-level governments and managerial units, can also be used to study individual healthcare facilities. It also related decision space to public and private sector facilities in Uganda’s formal referral system of healthcare and to different levels of capacity (i.e. from larger hospitals to smaller health centres) and determined associations between decision space and specific managerial functions. We found that, although Ugandan district-level entities are meant to have executive authority over personnel management, this was not seen for all personnel management functions. Namely, authority over discipline of staff seems to have devolved to be shared among both district- and facility-level personnel. However, district-level entities still maintain control over personnel management functions requiring greater human resources and financial investment—such as recruitment and promotion. This suggests administrative functions more subject to financial constraints are less subject to devolution of authority to lower administrative levels. We also saw higher drug-ordering autonomy for hospitals and level-IV health centres compared with level-III and -II health centres. This suggests that previous reforms to change Uganda’s medical supply-chain infrastructure to a hybrid ‘push-pull’ system have been adopted by a majority of public health facilities. Finally, we concluded that increased decision space may be associated with improved performance in some managerial functions, such as essential drug ordering, but not others, such as PM or QI. This difference may be due to factors such as knowledge of one’s own *de facto* decision space, ability to exercise decision-making autonomy in response to changes in local need, mechanisms of accountability for managerial performance, or facility capacity and access to resources. Future research is needed to both identify and create interventions to address such factors that mediate the relationship between decision space and specific managerial functions. We believe this is necessary for effective implementation of decentralization efforts, facilitating the development of strong healthcare management to meet the logistical challenges of ensuring equitable healthcare delivery for all.

## Supplementary Material

czab073_Supp

## Data Availability

The datasets supporting the conclusions of this article may be requested from the PMA 2020 repository (https://www. pma2020.org/request-access-to-datasets) managed and maintained by the Johns Hopkins Bloomberg School of Public Health, Department of Population, Family and Reproductive Health. The Stata codes used for data processing and analyses are available from the corresponding author on reasonable request.
