# Supplementary material for: Does decentralization of health systems translate into decentralization of authority? A decision space analysis of Ugandan healthcare facilities
Source: Health Policy Plan. 2021 Jun 24;36(9):1408–17. doi: 10.1093/heapol/czab074 (PMC8505862; doi:10.1093/heapol/czab074)
Supplement: czab073_Supp [file czab073_supp.zip › Appendix 1_Structure of O-GAP matrix.docx]

**APPENDIX 1.** Structure of the Opportunity-Score Matrix used during focus group discussions and average results.

| **OPPORTUNITY** | **LEVEL OF OPPORTUNITY**  **(1 year ago)** | | | | **LEVEL OF OPPORTUNITY (TODAY)** | | | | **CONTRIBUTION TO THE LEVEL OF OPPORTUNITY**  **BY THE INTERVENTION** | | | | **CONTRIBUTION TO THE LEVEL OF OPPORTUNITY**  **BY OTHER INITIATIVES** | | | | **LEVEL OF OPPORTUNITY (TODAY) WITHOUT THE INTERVENTION** | | | |
| --- | --- | --- | --- | --- | --- | --- | --- | --- | --- | --- | --- | --- | --- | --- | --- | --- | --- | --- | --- | --- |
|  | **What was the level of opportunity 1 years ago?**  Put 1 – 10 seeds (Where 1 is no opportunity and 10 is maximum opportunity)  Read carefully notes 1) and 2) | | | | **What is today the level of opportunity?**  Put 1 – 10 seeds (Where 1 is no opportunity and 10 is maximum opportunity)  Read carefully notes 1) and 2) | | | | **Has the intervention run contributed to the level of opportunity?**  if No put no seeds; if Yes ask: **in a positive or negative way?** Put with a post-it + for Positive and - for Negative; Then ask:  **How much positive/negative?** (1-10) (Where 1 is very little and 10 very much) | | | | **Have other initiatives/factors/actors contributed to the level of opportunity?**  if No put no seeds; if Yes ask: **in a positive or negative way?** Put with a post-it + for Positive and - for Negative; Then ask:  **How much positive/negative?** (1-10) (Where 1 is very little and 10 very much) | | | | **What would have been today the level of opportunity without the activity of CHF?**  Put 1 – 10 seeds (Where 1 is no opportunity and 10 is maximum opportunity)  Read carefully notes 1) and 2) | | | |
|  | *Poor hh part of group* | *Rich hh part of group* | *Poor hh not part of group* | *Rich hh not part of group* | *Poor hh part of group* | *Rich hh part of group* | *Poor hh not part of group* | *Rich hh not part of group* | *Poor hh part of group* | *Rich hh part of group* | *Poor hh not part of group* | *Rich hh not part of group* | *Poor hh part of group* | *Rich hh part of group* | *Poor hh not part of group* | *Rich hh not part of group* | *Poor hh part of group* | *Rich hh part of group* | *Poor hh not part of group* | *Rich hh not part of group* |
|  | Level 1/10 seeds | | | | Level 1/10 seeds | | | | sign + or - and 1/10 seeds | | | | sign + or - and contribution 1/10 seeds | | | | Level 1/10 seeds | | | |
| **To utilise health services when needed, without anxiety and impoverishment due to health expenditures** | **3** | **6** | **1** | **5** | **6** | **8** | **2** | **6** | **++++**  **+++** | **++++** | **++** | **=** | **+** | **+++** | **=** | **+++** | **4** | **7** | **1.5** | **6** |

NOTE that

1) every household is composed by husband and wife aged 25-45, three children aged 0-15, one elder aged 60-80;

2) households that are part of group have been participating actively in the pilot program for one year.
