# Supplementary material for: Does decentralization of health systems translate into decentralization of authority? A decision space analysis of Ugandan healthcare facilities
Source: Health Policy Plan. 2021 Jun 24;36(9):1408–17. doi: 10.1093/heapol/czab074 (PMC8505862; doi:10.1093/heapol/czab074)
Supplement: czab073_Supp [file czab073_supp.zip › Appendix 2_Robustness check alternative cut-off.docx]

|  | Catastrophic Health Expenditure (25% cut-off) | |  | Catastrophic Health Expenditure (10% cut-off) | |
| --- | --- | --- | --- | --- | --- |
|  |  |  | |  |  |
| ITT | -0.177*** |  | | -0.164 |  |
|  | (0.0454) |  | | (0.101) |  |
| HOUSEHOLD |  |  | |  |  |
| Cat He Exp 2019 (25% cut off) | 0.114*** |  | |  |  |
|  | (0.0393) |  | |  |  |
| Cat He Exp 2019 (10% cut off) |  |  | | 0.0594 |  |
|  |  |  | | (0.0494) |  |
| First wealth tertile (poor) | Ref |  | | Ref |  |
|  |  |  | |  |  |
| Second wealth tertile (average) | 0.0949 |  | | 0.116 |  |
|  | (0.0689) |  | | (0.0798) |  |
| Third wealth tertile (rich) | -0.0181 |  | | -0.0296 |  |
|  | (0.0638) |  | | (0.0982) |  |
| Other income source | -0.190*** |  | | -0.168*** |  |
|  | (0.0291) |  | | (0.0548) |  |
| Household size | -0.0121 |  | | -0.0145 |  |
|  | (0.0143) |  | | (0.0129) |  |
| Shocks | 0.00802 |  | | 0.0239 |  |
|  | (0.0137) |  | | (0.0149) |  |
| HOUSEHOLD HEAD |  |  | |  |  |
| Female HH head | 0.00772 |  | | -0.0413 |  |
|  | (0.0485) |  | | (0.0755) |  |
| Age HH head | 0.00366** |  | | 0.00367 |  |
|  | (0.00182) |  | | (0.00223) |  |
| Illiterate HH head | -0.0544 |  | | -0.0579 |  |
|  | (0.0636) |  | | (0.0951) |  |
| Constant | 0.237** |  | | 0.405** |  |
|  | (0.108) |  | | (0.172) |  |
|  |  |  | |  |  |
| Observations | 226 |  | | 226 |  |
| R-squared | 0.099 |  | | 0.076 |  |
| Note: Clustered robust SE in parentheses | |  | |  |  |
| *** p<0.01, ** p<0.05, * p<0.1 |  |  | |  |  |

**APPENDIX 2.** Comparison of ITT and ATET on Catastrophic Health Expenditure with alternative cut-offs.

|  | Catastrophic Health Expenditure (25% cut-off) |  | Catastrophic Health Expenditure (10% cut-off) |
| --- | --- | --- | --- |
|  |  |  |  |
| ATET | -0.218*** |  | -0.202* |
|  | (0.0546) |  | (0.118) |
| HOUSEHOLD |  |  |  |
| Cat. He. Exp. 2019 (25% cut off) | 0.114*** |  |  |
|  | (0.0384) |  |  |
| Cat. He. Exp. 2019 (10% cut off) |  |  | 0.0658 |
|  |  |  | (0.0469) |
| First wealth tertile (poor) | Ref |  | Ref |
|  |  |  |  |
| Second wealth tertile (average) | 0.105 |  | 0.126* |
|  | (0.0674) |  | (0.0760) |
| Third wealth tertile (rich) | 0.00821 |  | -0.00593 |
|  | (0.0576) |  | (0.0863) |
| Other income source | -0.187*** |  | -0.164*** |
|  | (0.0275) |  | (0.0522) |
| Household size | -0.0116 |  | -0.0142 |
|  | (0.0125) |  | (0.0121) |
| Shocks | 0.00239 |  | 0.0183 |
|  | (0.0127) |  | (0.0159) |
| HOUSEHOLD HEAD |  |  |  |
| Female HH head | 0.0351 |  | -0.0162 |
|  | (0.0484) |  | (0.0667) |
| Age HH head | 0.00327* |  | 0.00332 |
|  | (0.00169) |  | (0.00221) |
| Illiterate HH head | -0.0621 |  | -0.0656 |
|  | (0.0644) |  | (0.0929) |
| Constant | 0.257*** |  | 0.422** |
|  | (0.0993) |  | (0.175) |
|  |  |  |  |
| Observations | 226 |  | 226 |
| R-squared | 0.085 |  | 0.059 |
| Note: Clustered robust SE in parentheses | |  |  |
| *** p<0.01, ** p<0.05, * p<0.1 |  |  |  |
