# Supplementary material for: Does decentralization of health systems translate into decentralization of authority? A decision space analysis of Ugandan healthcare facilities
Source: Health Policy Plan. 2021 Jun 24;36(9):1408–17. doi: 10.1093/heapol/czab074 (PMC8505862; doi:10.1093/heapol/czab074)
Supplement: czab073_Supp [file czab073_supp.zip › Appendix 3_Differences for groups membership_rev.docx]

**APPENDIX 3.** Differences on observable characteristics related to community group membership.

|  | | **Mean** | | | **Difference** | **(SE)** |
| --- | --- | --- | --- | --- | --- | --- |
|  | | HH not part  of group | HH part  of group | |  |  |
| First wealth tertile (poor) | | 0.452 | 0.307 | | 0.145** | (0.063) |
| Second wealth tertile (average) | | 0.233 | 0.377 | | -0.144** | (0.063) |
| Third wealth tertile (rich) | | 0.315 | 0.311 | | 0.004 | (0.062) |
| Other income source | | 0.370 | 0.172 | | 0.198*** | (0.054) |
| Household size | | 4.068 | 5.734 | | -1.665*** | (0.279) |
| Shocks | | 3.288 | 3.422 | | -0.134 | (0.223) |
| Female HH head | | 0.301 | 0.139 | | 0.162*** | (0.050) |
| Age HH head | | 41.592 | 42.730 | | -1.139 | (2.006) |
| Illiterate HH head | | 0.260 | 0.164 | | 0.096* | (0.051) |
| *** p<0.01, ** p<0.05, * p<0.1 |  | | |  |  |  |
